# Hill Runner's Physiology, Performance and Nutrition: A Descriptive Study

**DOI:** 10.3389/fspor.2021.676212

**Published:** 2021-08-17

**Authors:** Liivia-Mari Lember, Thomas George Di Virgilio, Eilidh MacKenzie Brown, Nidia Rodriguez-Sanchez

**Affiliations:** ^1^Faculty of Natural Sciences, Department of Psychology, University of Stirling, Stirling, United Kingdom; ^2^Faculty of Health Sciences and Sport, Physiology, Exercise and Nutrition Research Group, University of Stirling, Stirling, United Kingdom

**Keywords:** endurance sport, body composition, anthropometry, VO_2_max, energy intake and expenditure, diet

## Abstract

**Objectives:** The aim of this descriptive study was to characterise anthropometric variables, aerobic capacity, running performance and energy intake and expenditure of hill runners in free-living conditions, and to investigate the relationship between age, anthropometric variables, aerobic capacity and running performance.

**Methods:** Twenty-eight hill runners participated in this study (17 males and 11 females; aged 18–65 years). Body fat percentage estimate, sum of eight skinfolds (triceps, subscapular, biceps, iliac crest, supraspinale, abdominal, front thigh and medial calf) and maximal oxygen capacity (VO_2_max) were assessed in a laboratory setting. Participants also completed a timed hill run (Dumyat Hill, Scotland, ascent: 420 m, distance: 8 km) while wearing a portable gas analyzer to assess oxygen consumption (VO_2_). Energy intake and energy expenditure were assessed in free-living conditions over three consecutive days different from the testing days through self-reported food diaries and accelerometers.

**Results:** VO_2_max assessed in the lab (51.2 ± 7.6 ml·min^−1^·kg^−1^) showed a weak negative relationship with age [rs(23) = −0.38, *p* = 0.08]. Neither body fat percentage (median 12.4; IQR 10.1–17.1) nor the sum of skinfolds (median 81.8; IQR 62.4–97.8 mm) correlated with age [rs(28) = 0.001, *p* = 0.10 and 26 rs(28) = −0.02, *p* = 0.94, respectively]. The observed intensity of the hill run was 89 ± 6% of the age predicted maximum heart rate and 87 ± 9% of the VO_2_max observed in the lab. Hill running performance correlated with VO_2_max [r(21) = 0.76, *p* < 0.001], age [rs(26) = −0.44, *p* = 0.02] and with estimated body fat percentage and sum of skinfolds [rs(26) = −0.66, *p* < 0.001 and rs(26) = −0.49, *p* = 0.01, respectively]. Energy intake negatively correlated with age [rs(26) = −0.43, *p* = 0.03], with the overall energy intake being significantly lower than the total energy expenditure (2273 ± 550 vs. 2879 ± 510 kcal·day^−1^; *p* < 0.001; *d* = 1.05).

**Conclusion:** This study demonstrated that hill running performance is positively associated with greater aerobic capacity and negatively associated with increases in adiposity and age. Further, the study highlights that hill runners are at risk of negative energy balance.

## Introduction

Hill running is an endurance sport where intensity and duration are influenced by environmental factors such as terrain and weather and by runners' fitness and nutrition. Endurance athletes such as ultramarathon runners have been observed healthier with lower incidence of injuries and illnesses when compared to the general population (Hoffman and Krishnan, 2014) however, exercising in a mountainous and rocky landscape may increase the risk of falls and injuries as a result of respiratory and locomotor muscle fatigue (Tiller, 2019) highlighting the importance of runners' fitness and nutrition. While the demographics of hill runners are unknown, evidence suggests that running and hill walking are both favoured sports among older adults (Ainslie et al., 2002; Stevinson and Hickson, 2014; Lepers and Stapley, 2016). Therefore, understanding the intensity of hill running and how physiological factors such as body composition and maximal oxygen capacity (VO_2_max) influence hill running performance across different ages can help improve athletic performance and indicate whether hill running promotes healthy ageing.

Population demographics are changing considerably, with older adults expected to account for over 16% of the global population by 2050 (Tanaka and Seals, 2008; Nations et al., 2019). With this increase comes a rise in the number of masters athletes (>40 years old) who are often referred to as examples of “exceptionally successful ageing” (Tanaka and Seals, 2008). Ageing is commonly associated with a decline in the body's capacity for physical activity, with previous research highlighting a decrease in VO_2_max as an indicator of this decline (Grimby et al., 1966). Reduction in aerobic capacity, however, has been linked to a decline in running performance (Fornasiero et al., 2018). Moreover, VO_2_max is also a strong predictor of cardiovascular and all-cause mortality (Valenzuela et al., 2020). It is estimated that VO_2_max in the general population decreases by around 10% per decade after the age of 30 (Robinson, 1938), largely due to reductions in maximal heart rate and stroke volume (Heath et al., 1981; Rodeheffer et al., 1984). Nonetheless, research suggests that an active lifestyle can slow such reductions in cardiovascular function: in a physically active population VO_2_max declines by an estimated 5% per decade (Hagberg, 1987). Moreover, older adults often experience body composition alterations in the absence of fluctuations in weight or body mass index. These changes are likely due to increases in fat mass, alongside reductions in lean muscle mass and bone mineral density (St-Onge, 2005). Excess adiposity in turn has been demonstrated to negatively influence athletic performance due to greater muscular effort required to accelerate the extra weight (Legaz and Eston, 2005; Fornasiero et al., 2018). Exercising however, can prevent increases in age induced body fat (Piasecki et al., 2016, 2019) and reductions in lean mass (Piasecki et al., 2019) further emphasising the potential benefits of hill running.

Although running can aid healthy ageing by decelerating decline in aerobic capacity, loss of lean mass and increase in body fat (Heath et al., 1981; Piasecki et al., 2016), it is noteworthy that endurance athletes are at risk of energy deficits (Mountjoy et al., 2018). Furthermore, it is thought that 15–30% of older adults have “anorexia of ageing” (Malafarina et al., 2012), a condition whereby individuals experience a reduction in appetite leading to reduced nutrient intake (Morley and Silver, 1988; Payette et al., 1995). For those who continue to engage in regular physical activity into middle and old age, greater energy expenditure relative to energy intake can lead to a negative energy balance. Prolonged negative energy balance can in turn lead to weight loss, and in some cases to impaired immunity, protein synthesis, cardiovascular and bone health (Mountjoy et al., 2018). The high-energy requirements associated with exercising in a mountainous environment (Ainslie et al., 2005; Rodríguez-Marroyo et al., 2018), uphill running (Gostill et al., 1974; Staab et al., 1992) and eccentric exercise occurring during downhill running (Gostill et al., 1974; Paschalis et al., 2011) may compound such impairments, placing older adults at a greater overall risk of adverse health effects. Therefore, observing energy intake in relation to expenditure can provide insight into whether hill running poses a risk of negative energy balance. This is particularly important as inadequate energy intake can lead to fatigue-induced injuries, especially when exercising on varying terrain and in mountainous environments (Ainslie et al., 2005; Tiller, 2019).

Despite the popularity of hill running there is a dearth of evidence on the physiological and metabolic implications of participating in this sport. Therefore, the aim of this descriptive study was to characterise anthropometric variables, aerobic capacity and hill running performance in hill runners from different ages and to investigate the relationship between age, anthropometric variables, aerobic capacity and running performance. A further aim was to observe energy intake and expenditure in free-living conditions in hill runners. We expected to find a decline in aerobic capacity and an increase in adiposity with age that would negatively impact the hill running performance. Furthermore, we hypothesised that energy intake would decrease with age.

## Materials and Methods

### Participant Characteristics and Ethical Approval

Twenty-eight healthy and active recreational hill runners (17 males and 11 females; aged 18–65 years; median: 41 years) were recruited from local clubs for the study. All participants self-identified as hill runners as opposed to road, track, cross-country or any other running discipline athletes. Participants were considered hill runners if they (1) self-identified as hill runners and (2) regularly participated in hill running. All but one participant reported participating in hill racing competitions. Participation in the study was voluntary and written informed consent was obtained prior to testing. The study was approved by the NHS, Invasive or Clinical Research (NICR) Ethics Committee of the University of Stirling (project reference number: NICR (18/19) Paper 020) and adhered to the principles set out by the Declaration of Helsinki (2013).

### Study Design

This study employed a between participants, cross-sectional design. Participants attended to the University of Stirling facilities in two occasions: once for a lab-based test and the second time for a field-based testing session, with a minimum of 72 h between testing days. On both occasions, participants arrived following an overnight fasting period (≥10 h), having consumed no alcohol or caffeine for a minimum of 12 h prior to testing. Participants were also instructed to arrive in a rested state having refrained from physical exercise for 24 h prior. Anthropometric measurements and maximal fitness test were carried out in the laboratory visit. Self-reported hill running participation history (years) and hill running training frequency (hours per week) information was also collected. Field session involved a timed run up and down a local hill (Dumyat, ascent: 440 m, maximum elevation per km: 117 m, mean elevation per km ascent: 91.3 m, run distance: 8 km) while wearing a portable gas analyzer (see below). Participants were also provided with a food and fluid diary to complete and an accelerometer (ActiGraph GT3X, Pensacola, USA) to wear over three consecutive days (one weekend day) separate from the testing session days.

### Laboratory Testing

#### Anthropometric Measurements

Anthropometric measurements (height, body mass, skinfolds, girths and breadths) were taken in the morning of the lab test day in accordance with the International Society for the Advancement of Kinanthropometry (ISAK) standards by two accredited level 1 anthropometrists (Stewart and Marfell-Jones, 2011).

Participants were barefoot and wearing minimal clothing during measurements. Height was recorded using a stadiometer (Marsden HM-250P, Rotherham, UK) and body mass using an electronic set of scales (Seca 804, Hamburg, Germany). Skinfold thicknesses were measured from the right side of the body from triceps, subscapular, biceps, iliac crest, supraspinale, abdominal, thigh and medial calf sites using Harpenden skinfold callipers (HaB International Ltd., Warwickshire, UK). Two measurements were taken from each site unless an intra-measurer target of ≤ 5% for skinfolds and ≤ 1% for other measurements was breached, in which case a third measurement was taken (Stewart and Marfell-Jones, 2011). For data analysis the mean of 2 measurements or the median of 3 measurements was used (Stewart and Marfell-Jones, 2011).

Somatotypes were determined following Carter and Heath method (Stewart and Sutton, 2012). Body fat percentage (BF%) was calculated using Withers et al. (1987a,b) equations developed for athletic population.

#### Maximal Aerobic Capacity

Laboratory-assessed maximal aerobic capacity (VO_2_max) was measured during an incremental treadmill test (adapted from Wiswell et al., 2000) whilst participants wore a portable gas analyzer (Oxycon Mobile, Jaeger, Würzburg, Germany). All participants completed a warm-up consisting of walking or jogging. The testing protocol started at 8 km · h^−1^ and 0% incline, then alternated increases in speed and incline by 2 km · h^−1^ and 2% every 3 min until volitional exhaustion (protocol available in Supplementary Table 1). Participants rated their perceived exertion using the Borg Scale (Borg and Noble, 1974) at the end of each stage until exhaustion. Heart rate (HR) was recorded using a chest heart rate sensor (Polar H1, Kempele, Finland) for 12 participants and with a wrist heart rate monitor (Garmin Forerunner 30, Garmin Ltd., USA) for 16 participants due to technical difficulties. Polar chest strap monitor and wrist-worn Garmin Forerunner have both demonstrated high agreement with electrocardiogram on treadmill-based exercise (*r*_c_ = 0.99 and 0.92, respectively) (Gillinov et al., 2017).

Gas analyses were performed using a pre-calibrated breath by breath ergospirometry device recording 8-breath means for both testing sessions to allow for comparison between the laboratory and field VO_2_ values. VO_2_ and HR data (from chest HR monitor) were analysed using LabManager software (V5.3.0, Cardinal Health, USA) generating a report of 1-min means.

VO_2_max was considered attained when VO_2_ plateaued. Plateau in VO_2_ was considered achieved when the change in average VO_2_ between stages decreased to less than half of the normal stage-to-stage difference in VO_2_ [adapted from Hogg et al. (2015)] and when the difference was ≤ 2.1 ml · kg^−1^ ·min^−1^ (Midgley et al., 2007). Since the incidence of plateau using 1-min means is lower than with shorter breath-by-breath sampling rates (Astorino, 2009) a secondary criterion was accepted for determining the attainment of VO_2_max when plateau in VO_2_ was not reached. VO_2_max was considered achieved when two of the following criteria were met: (1) RER ≥ 1.05, (2) HR ± 10 bpm of age-predicted maximum (maximum HR equating 220 minus the age of the participant) and (3) RPE ≥ 17 [adapted from Wiswell et al. (2000); Hogg et al. (2015)]. RPE rating was collected in the end of each stage of the VO_2_max protocol and thus, RPE rating is missing for eight participants who reached exhaustion before the conclusion of the stage. For those participants, a conservative approach was utilised by using the RPE rating from the previous completed stage for VO_2_max determination.

### Field Testing

For the field run test, participants were fitted with the previously described pre-calibrated portable gas analyser, validated for prolonged field testing in windy, humid and low temperature conditions (Salier Eriksson et al., 2012), a chest HR sensor (Polar H1, Kemple, Finland) and a GPS watch (Garmin Forerunner 30, Garmin Ltd., USA). Participants were instructed to run as they usually would. Running route was marked with tape and participants were given both written and verbal instructions prior to setting off (route map available in Supplementary Figure 1). One of the researchers was at the top of the hill to verify each runner's wellbeing and the completion of the route. Regardless of the researchers' efforts a couple of participants deviated slightly from the signalised round, and due to these deviations pace was used in the analyses by dividing distance travelled (km) by time (h). Average VO_2_, RER and HR were calculated per km for each participant based on their average pace. Average values for each km were determined if ≥50% of data for that km were available (slight deviations in trajectories meant that some participants ran <8 km, whereas for some participants the recording failed prior to the conclusion of the run). For the participants who ran more than 8 km data for the additional distance was not included in the average VO_2_, RER and HR per km analyses.

### Dietary Intake and Energy Expenditure

Energy intake (EI) and total energy expenditure (TEE) were estimated and analysed over three consecutive days (including one weekend day; not overlapping with the testing sessions) in free-living conditions.

Energy intake was estimated through a self-reported weighed food and fluid diary. Participants were encouraged to follow their usual dietary habits and were instructed to provide detailed description, including the name, brand, weight, cooking method as well as the weight of leftovers for all food and drink consumed. If required, participants were provided with electronic kitchen scales (CS 200E, Ohaus Corp., USA). Energy intake and macronutrient composition were analysed using a dietary analysis software (Nutritics Ltd., Ireland). Missing foods were manually entered to the database by referring to food labels. Participants were contacted for clarification in case of ambiguous information.

Active energy expenditure (AEE) was recorded using a triaxial accelerometer (see above) strapped around the waist using an elastic belt (Hwang et al., 2018). Participants were instructed to wear the accelerometer during waking hours (≥8 h · day^−1^) except when showering or swimming.

Accelerometer data was downloaded using ActiLife software (v6. 13.3, ActiGraph LLC., USA) using Williams Work-Energy (1998) algorithm for energy expenditure and Freedson Adult VM3 (2011) cut points (Sasaki et al., 2011). One second sampling epochs were recorded at a 30 Hz sample rate (Hwang et al., 2018).

Resting metabolic rate (RMR) was calculated using Cunningham's equation (Thompson and Manore, 1996). Predicted total energy expenditure (TEE) was calculated by adding AEE to RMR and dividing the result by 0.9 to account for the diet induced thermogenesis (Westerterp, 2004).

### Statistical Analyses

Hill running participation data are missing for two participants due to measurement error (years running *n* = 2; hours running *n* = 1). HR data from VO_2_max are missing for five participants due to measurement error. Twenty seven out of 28 participants completed the hill run (one participant dropped out due to illness). Hill run pace data are missing for one participant due to measurement error. Hill running VO_2_ and RER data are missing for six and incomplete (11–87% data available) for seven participants due to equipment failure. Hill running HR data are missing for 10 and incomplete (73–94% data available) for four participants due to technical difficulties. Energy intake data are missing for two participants and expenditure data for one participant due to drop out.

All tests were carried out using jamovi (jamovi v 1.6.7.0, www.jamovi.org). Data were tested for normality using the Shapiro-Wilk test. Endomorphy and ectomorphy data were not normally distributed, so the difference in somatotype scores was tested with Friedman test and followed up with Wilcoxon rank tests to determine the source of significance. Relationships between non-normally distributed variables were assessed using Spearman's correlation coefficients and using Pearson's correlation coefficients for normally distributed data. Coefficients were interpreted as previously described by Evans (Evans, 1996): 0.00–0.19 = very weak; 0.20–0.39 = weak; 0.40–0.59 = moderate; 0.60–0.79 = strong; 0.80–1.0 = very strong. Difference between energy intake and total energy expenditure were analysed using two-tailed paired *t*-test. Effect size was quantified using Cohen's d and interpreted as: 0.2 = small; 0.5 = medium; 0.8 = large (Cohen, 1988). Normally distributed data are presented as means ± SD and non-normally distributed data are presented as median and IQR (Habibzadeh, 2017). Statistical significance was set as *p* ≤ 0.05 for all statistical tests.

## Results

### Descriptive Characteristics

Participant characteristics are presented in Table 1. Participants had been hill running for a median of 5 years (IQR 3–10) and were hill running on average 3.8 ± 2.0 h per week. The association between age and hill running experience was not statistically significant [rs(26) = 0.26, *p* = 0.21]. Further, the relationships between age and estimated body fat percentage (median 12.4; IQR 10.1–17.1) and sum of eight skinfolds (median 81.8; IQR 62.4–97.8 mm) were statistically negligible [rs(28) =0.001, *p* = 0.10 and rs(28) = −0.02, *p* = 0.94, respectively]. Significant relationships were observed between endomorphy, mesomorphy and ectomorphy scores [X^2^(2) = 19.0, *p* < 0.001]. Specifically, participants were significantly more mesomorphic than endomorphic and ectomorphic [*p* < 0.001 for both], whereas there was no significant difference between endomorphy and ectomorphy [*p* = 0.84].

**Table 1:** Participant characteristics.

	***n***	**Mean ± SD**
Age (y)^*^	28	41 (27–52)
Running experience (y)^*^	26	5 (3–10)
Running hours (h·week^−1^)	27	3.8 ± 2.0
Body mass (kg)	28	67.8 ± 11.4
Height (m)	28	1.74 ± 0.11
Σ8 skinfolds (mm)^*^	28	81.8 (62.4–97.8)
Body fat (%)^*^	28	12.4 (10.1–17.1)
Endomorphy^*^	28	2.7 (2.1–3.3)
Mesomorphy	28	4.3 ± 0.9
Ectomorphy^*^	28	2.9 (1.9–3.7)
VO_2_max (ml·min^−1^·kg^−1^)	23	51.2 ± 7.6
RER at VO_2_max	23	1.05 ± 0.05
HR at VO_2_max (bpm)	23	171 ± 18
RPE at VO_2_max^*^	23	18 (17–19)
VO_2_peak (ml·min^−1^·kg^−1^)	5	52.3 ± 5.9
RER at VO_2_peak	5	1.05 ± 0.07
HR at VO_2_peak (bpm)	5	170 ± 17
RPE at VO_2_peak	5	17 ± 3
Running performance (km·h^−1^)	26	8.8 ± 1.6
VO_2_ (ml·min^−1^·kg^−1^)	21	44.3 ± 6.7
RER	21	0.80 ± 0.05
HR (bpm)	17	159 ± 13

* *Data are displayed as median (IQR). Σ8 skinfolds = sum of triceps, subscapular, biceps, iliac crest, supraspinale, abdominal, front thigh and medial calf skinfolds*.

### Aerobic Capacity

**Figure 1:**
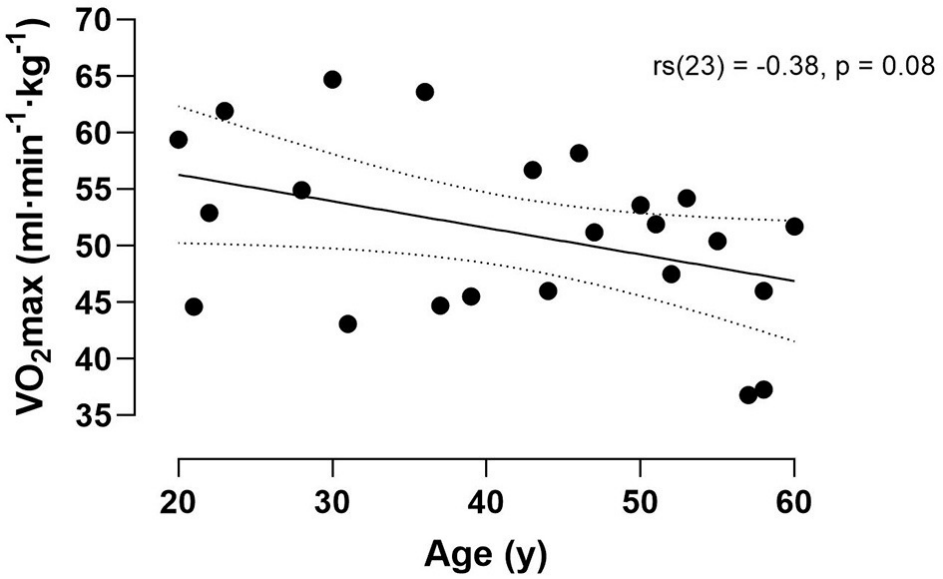
Correlation between VO_2_max and age. Dotted lines denote 95% CI.

Of the 28 participants 23 met the VO_2_max criterion (51.2 ± 7.6 ml·min^−1^·kg^−1^). For the remaining five participants a peak VO_2_ was established (52.3 ± 5.9 ml·min^−1^·kg^−1^). Maximal aerobic capacity assessed in the lab showed a trend for a negative relationship with age [rs(23) = −0.38, *p* = 0.08] (Figure 1).

### Hill Running Performance

**Figure 2:**
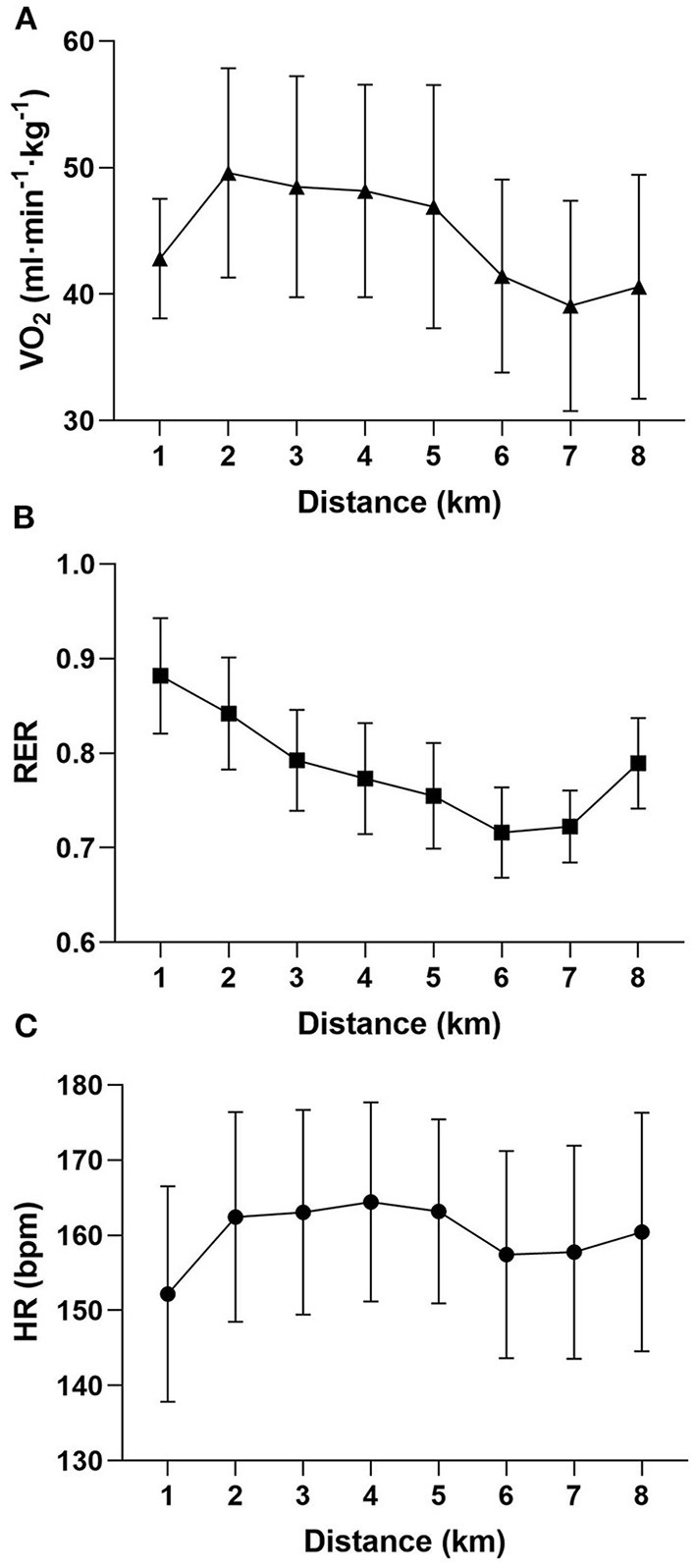
Average VO_2_ **(A)**, RER **(B)** and HR **(C)** values per km of hill run (*n* = 16). Error bars denote SD.

The average field test running speed was 8.8 ± 1.6 km·h^−1^. During the run, HR was 159 ± 13 bpm, representing 89 ± 6% of the age predicted maximum HR. VO_2_ during the run was 44.4 ± 6.7 ml·min^−1^·kg^−1^, constituting 87 ± 9% of the VO_2_max observed in the lab. RER during the hill run was on average 0.80 ± 0.05. VO_2_, RER, and HR per km of hill run are shown in Figure 2.

**Figure 3:**
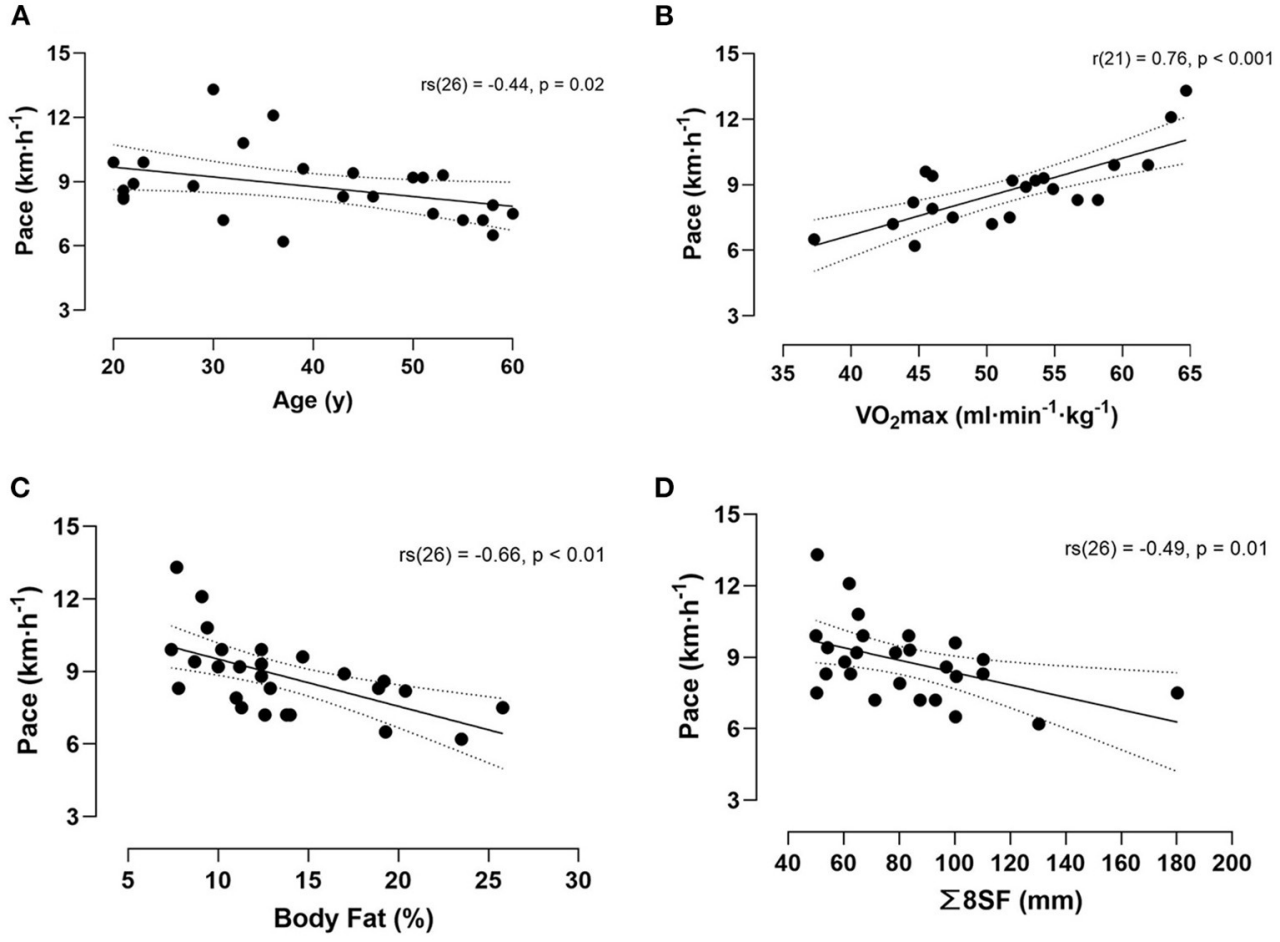
Correlation between the running performance and age **(A)**, VO_2_max **(B)**, body fat percentage **(C)** and sum of eight skinfolds **(D)**. Dotted lines denote 95% CI.

Hill running performance showed a negative moderate correlation with age [rs(26) = −0.44, *p* = 0.02] (Figure 3), a moderate correlation with training frequency (hours hill running per week) [r(25) = 0.43, *p* = 0.03] and a weak correlation with years hill running [rs(24) = 0.35, *p* = 0.09].

Both estimated body fat percentage and sum of eight skinfolds negatively correlated with running performance [rs(26) = −0.66, *p* < 0.001 and rs(26) = −0.49, *p* = 0.01, respectively] (Figure 3). Mesomorphy and endomorphy also negatively correlated with running performance [r(26) = −0.21, *p* = 0.31 and rs(26) = −0.70, *p* < 0.001, respectively], whereas ectomorphy was positively associated with performance [rs(26) = 0.48, *p* = 0.01].

Finally, there was a strong significant relationship between VO_2_max and performance [r(21) = 0.76, *p* < 0.001] (Figure 3).

### Dietary Intake and Total Energy Expenditure

**Figure 4:**
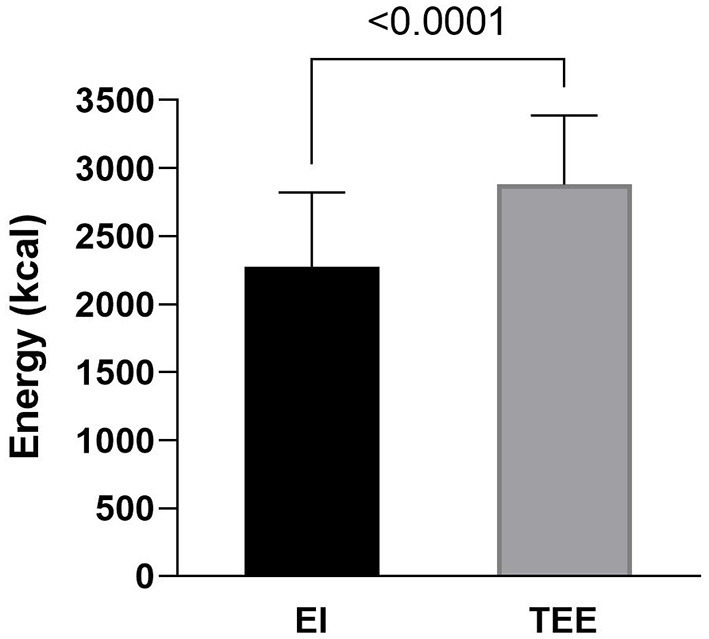
Three-day average energy intake (EI) (*n* = 26) and total energy expenditure (TEE) (*n* = 27). Error bars denote SD.

**Figure 5:**
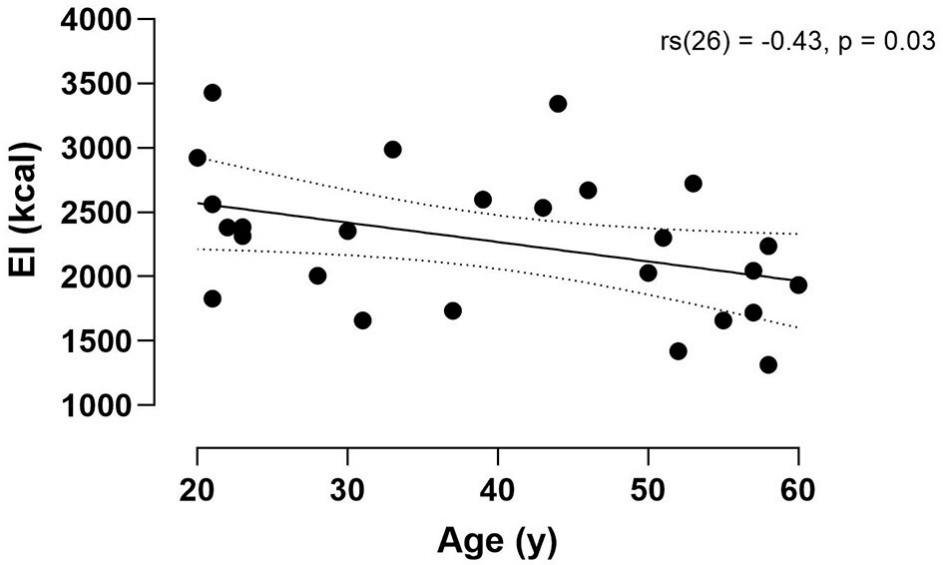
Correlation between 3-day average energy intake (EI) and age. Dotted lines denote 95% CI.

Average daily energy intake (2273 ± 550 kcal·day^−1^) was significantly lower than total energy expenditure (2879 ± 510 kcal·day^−1^) [*t*(25) = 5.37, *p* < 0.001; *d* = 1.05] (Figure 4). Moreover, there was a significant moderate negative relationship between age and average energy intake [rs(26) = −0.43, *p* = 0.03] (Figure 5). Whereas, the relationship between age and total energy expenditure was not statistically significant [rs(26) = −0.17, *p* = 0.39].

The macronutrient intake was 4.2 ± 1.5 g·kg^−1^·day^−1^ for carbohydrates, 1.4 ± 0.6 g·kg^−1^·day^−1^ for protein and a median of 1.1 (IQR 1.0–1.6) for fat. On average, carbohydrates contributed to 47 ± 7%, fat 34 ± 6% and protein a median of 14% (IQR 13–17%) of the daily total energy intake.

## Discussion

This study described the body composition, aerobic capacity, performance, dietary intake and energy expenditure of hill runners across different ages. The findings demonstrated: (1) decline in aerobic capacity with age; (2) no relationship between age and adiposity; (3) negative association between hill running performance and (a) decrease in aerobic capacity, (b) increase in adiposity and (c) increase in age; (4) significantly lower energy intake than total energy expenditure in free living conditions and a decrease in energy intake with increase in age.

Aerobic capacity is estimated to decline by 5% per decade in athletes (instead of the 10% decrease observed in sedentary people) (Robinson, 1938; Åstrand, 1968; Hagberg, 1987). Whilst we observed a negative trend between VO_2_max and age this relationship was not strong. This finding is of interest as it suggests that hill running participation may be beneficial in maintaining cardiorespiratory fitness. In contrast, a meta-analysis assessing VO_2_max in endurance athletes showed more drastic declines in older men when compared to their younger counterparts (Wilson and Tanaka, 2000). Participants in the current study identified themselves as hill runners, the potentially different physiological and metabolic demands of hill running compared to endurance sports could explain why the decline in VO_2_max was not as evident as in previous literature. Nonetheless, it is noteworthy that hill running performance was significantly associated with age and aerobic capacity in this study. It has been reported that most endurance athletes experience a decrease in their athletic performance with increase in age, including a reduction in cardiovascular function due to a loss of muscle mass (Fleg and Lakatta, 1988), a lower maximal heart rate, cardiac output, arteriovenous oxygen differences and maximal aerobic capacity (Fleg and Lakatta, 1988; Wilson and Tanaka, 2000; Pantoja et al., 2016; Willy and Paquette, 2019; Valenzuela et al., 2020). However, a longitudinal observation of endurance runners spanning over five decades demonstrated that training and racing may limit the reduction in performance to 7% per decade (Lepers et al., 2021). Taken together, the present findings suggest that hill running may be beneficial for maintaining aspects of cardiorespiratory fitness that would otherwise be negatively affected by ageing. Nonetheless, as observed in other endurance sports (Lepers and Stapley, 2016), hill running performance declined with increase in age in this cohort potentially due to a combination of factors including reduction in aerobic and muscular capacity.

Ageing is associated with alterations in body composition and body fat distribution (Pararasa et al., 2015): adipose tissue increases while lean muscle mass (Sakuma and Yamaguchi, 2010) and bone mineral density decrease (Tomlinson et al., 2019). Interestingly, in this study no associations were observed between the sum skinfold thicknesses and the estimated body fat percentage with an increase in age. Similar findings have previously been observed in endurance-trained men (Wilson and Tanaka, 2000). Previous literature suggests that increased body fat negatively affects running performance (Legaz and Eston, 2005; Fornasiero et al., 2018; Herrmann et al., 2019). In our sample we found that running performance was significantly decreased with an increase in age and adiposity, however, the findings suggests that the “worse” performance in older runners was possibly mediated by factors other than adiposity. Further, hill runners in our study were predominantly mesomorphic. It could be expected that hill runners develop a more muscular physique due to increased activation of leg muscles during uphill running compared to level running (Vernillo et al., 2017) however, interestingly both meso-and endomorphy demonstrated negative relationships with running performance. Mesomorphy in the current study sample was not necessarily an adaptation to increased muscular recruitment during hill running. While uphill running has demonstrated increased activation of the vastus group and soleus, other muscles such as rectus femoris, gracilis and semitendinosus have demonstrated reduced activation during uphill running when compared to level running (Sloniger et al., 1997). Moreover, research investigating muscular activation during hill running has not accounted for the reduction of speed during uphill running (Vernillo et al., 2017). In our sample, only ectomorphy was positively associated with hill running performance. These results are consistent with previous research suggesting that lower body mass promotes performance due to lower muscular effort required for running (Vernillo et al., 2013).

Ageing is associated with a reduction in appetite and food intake resulting in a decline in energy intake of ~1% per year, this effect is termed the anorexia of ageing (Chapman et al., 2002). Moreover, Louis et al. (2020) has identified some of the metabolic challenges ageing athletes face such as the risk of low energy availability and anabolic resistance. In addition, the energy cost of running can be expected to be higher for older runners (Pantoja et al., 2016). Participating in physical activity is thought to increase appetite, reducing the risk of the anorexia of ageing; however, a recent systematic review concluded that there is insufficient evidence to apply this statement to older adults (Clegg and Godfrey, 2018). This is supported by our findings: reduced energy intake in the current study was significantly associated with age, suggesting that an active lifestyle may not be sufficient to moderate reduction in appetite associated with ageing. Moreover, the current study results demonstrate a lower EI in comparison to TEE in free-living conditions in hill runners. Further, participants in the present study did not meet the recommended carbohydrate intake although protein intake was within the recommended range (Thomas et al., 2016; Louis et al., 2020). Our findings in hill runners support what the International Olympic Committee energy availability consensus reports: endurance athletes are at increased risk of negative energy availability (Mountjoy et al., 2018). Prolonged negative energy availability can, however, negatively influence several physiological functions and sports performance which in mountainous environment may also contribute to increased risk of injury (Ainslie et al., 2005). Further highlighting the importance of sufficient energy intake for hill runners is the high intensity of hill running observed in this study despite participants being instructed to run as they would normally. Participants were running at an intensity of 89 ± 6% of the age predicted maximum HR and 87 ± 9% of the VO_2_max observed in the lab; similar to values previously reported in longer distance mountain races (Rodríguez-Marroyo et al., 2018). Regardless, the average RER (0.80 ± 0.05) observed during the hill run suggests that runners were primarily metabolising fats, although, the transferability of this finding should be interpreted with caution considering that participants were running in a fasted state.

This study has some limitations. The study was conducted in a relatively small sample (*n* = 28) using a cross-sectional design. Future research with a larger sample size is required to confirm the current observations. Further, energy and macronutrient intake relied on self-report food records; even though participants received detailed instructions on filling them by the researchers, there is a possibility of participants reporting erroneous information. However, considering the limited data on hill running discipline, we believe it is an important start point for further research on hill runners. Another limitation is that body composition and energy expenditure were estimated through anthropometric measurements and triaxial accelerometers, respectively; using more accurate methods (e.g., dual energy X ray absorptiometry, doubly labelled water) could reduce the possibility of errors. Future research could also incorporate measurements of strength and muscle function to obtain further insight regarding the neuromuscular and biomechanical responses to hill running.

## Conclusion

The outcomes from this study characterise the intensity of hill running and highlight that hill runners are at risk of negative energy balance in free-living conditions. Limited previous research in hill runners and the relatively small sample size in the current study precludes a meaningful inference of the current findings and thus, warrants further investigation using control groups of inactive participants or runners from other disciplines. Regardless, the present study shows that hill running performance is positively associated with greater aerobic capacity and negatively associated with increases in adiposity and age. Further, the findings of the present study emphasise the importance of activities such as hill running to decelerate age induced declines in maximal aerobic capacity and increases in body fat.

## Data Availability Statement

The original contributions presented in the study are included in the article/Supplementary Material, further inquiries can be directed to the corresponding author/s.

## Ethics Statement

The studies involving human participants were reviewed and approved by NHS, Invasive or Clinical Research (NICR) Ethics Committee of the University of Stirling. The patients/participants provided their written informed consent to participate in this study.

## Author Contributions

NR-S, L-ML, and EB designed the study. L-ML and EB performed data collection. TD and L-ML performed the statistical analysis. L-ML, TD, and NR-S wrote the manuscript with EB. All authors contributed to the article and approved the submitted version.

## Conflict of Interest

The authors declare that the research was conducted in the absence of any commercial or financial relationships that could be construed as a potential conflict of interest.

## Publisher's Note

All claims expressed in this article are solely those of the authors and do not necessarily represent those of their affiliated organizations, or those of the publisher, the editors and the reviewers. Any product that may be evaluated in this article, or claim that may be made by its manufacturer, is not guaranteed or endorsed by the publisher.
