# Supplementary material for: Hill Runner's Physiology, Performance and Nutrition: A Descriptive Study
Source: Front Sports Act Living. 2021 Aug 17;3:676212. doi: 10.3389/fspor.2021.676212 (PMC8415831; doi:10.3389/fspor.2021.676212)
Supplement: Supplementary file 1 [file Data_Sheet_1.pdf]

Supplementary Material

Supplementary Table 1. VO_2max testing protocol.

Stage	Time (min)	Velocity (km · h⁻¹)	Incline (%)
1	0 - 3	8	0
2	3 - 6	8	2
3	6 - 9	10	2
4	9 - 12	10	4
5	12 - 15	12	4
6	15 - 18	12	6
7	18 - 21	14	6
8	21 - 24	14	8
9	24 - 27	16	8
10	27 - 30	16	10
11	30 - 33	16	12

A

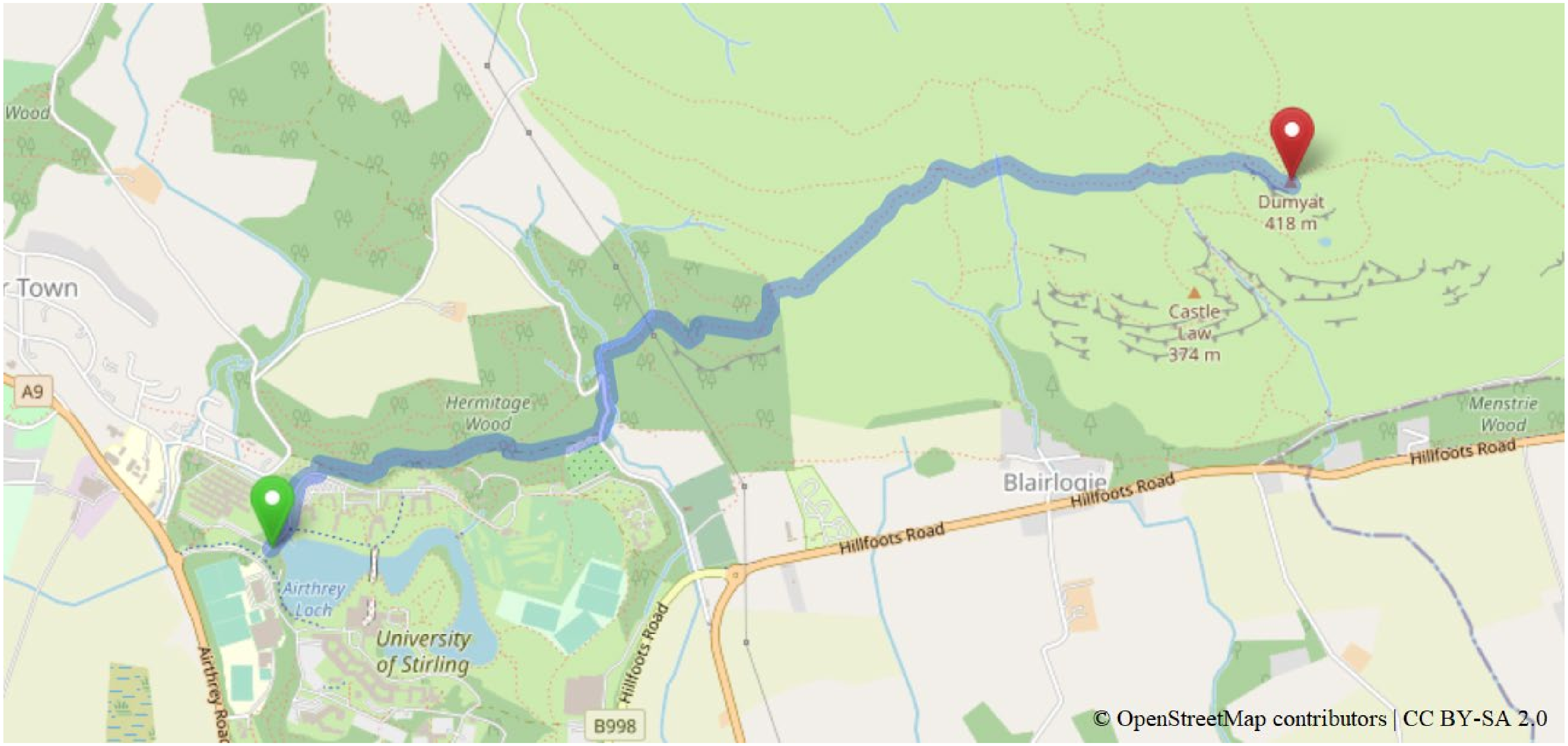
Wood
Town
A9
Hermitage Wood
Airthrey Loch
University of Stirling
Airthrey Road
Hillfoots Road
B998
Dumyat 418 m
Castle Law 374 m
Blairlogie
Hillfoots Road
Menstrie Wood
Hillfoots Road
© OpenStreetMap contributors | CC BY-SA 2.0

B

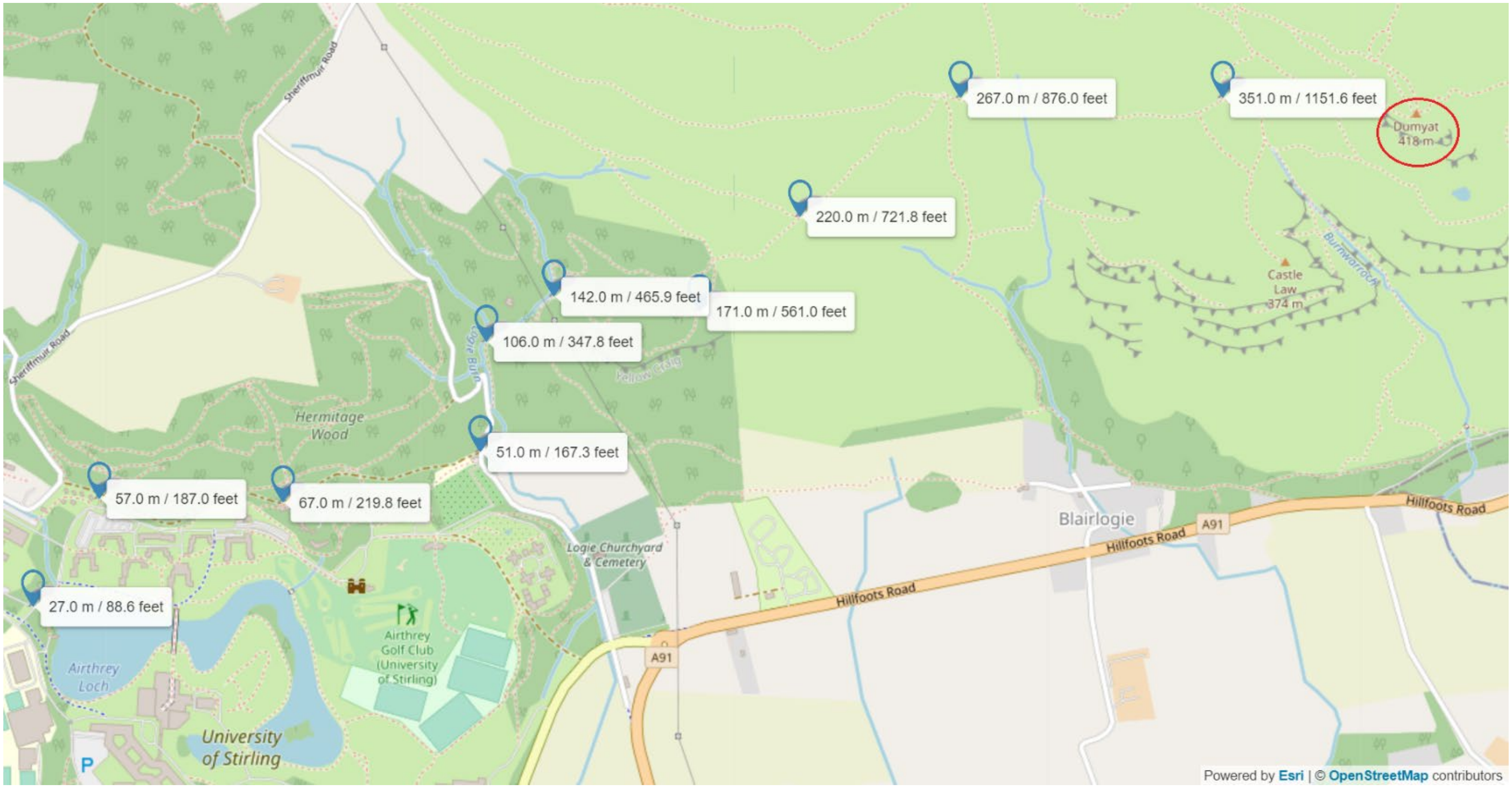

Supplementary Figure 1. (A) Dumyat run route; start and finish point (green marker on the map) is located at the cross of Pathfoot Road and Hermitage Road, Stirling, UK. (B) Altitude points of the Dumyat run route. Run route map (A) is generated and accessible via

www.openstreetmap.org and altitude points are generated using © Free Map Tools, accessible via https://www.freemaptools.com/elevation-finder.htm.
